# Optimizing Study Design for Evaluating Complex Interventions: An Example of a Feasibility Study in Person-Centered and Integrated Chronic Disease Care in Dutch General Practice

**DOI:** 10.5334/ijic.8998

**Published:** 2025-11-06

**Authors:** Lena H. A. Raaijmakers, Tjard R. Schermer, Hester E. van Bommel, Jan H. Vercoulen, Tessa van Loenen, Erik W. M. A. Bischoff

**Affiliations:** 1 Department of Primary and Community Care, Radboud University Medical Center, Research Institute for Medical Innovation, Nijmegen, The Netherlands; 2 Gelre Hospitals, Science Support Office, Apeldoorn, The Netherlands; 3 Pharos, Dutch Centre of Expertise on Health Disparities, Utrecht, The Netherlands; 4 Department of Medical Psychology, Radboud University Medical Center, Radboud Institute for Health Sciences, Nijmegen, The Netherlands; 5 Department of General Practice, Erasmus University Medical Centre, Rotterdam, The Netherlands

**Keywords:** person-centred integrated care, multimorbidity, chronic disease management, general practice

## Abstract

**Background:**

Complex interventions are frequently used at different levels in healthcare. The main aim of this paper is to describe a method for conducting a feasibility study in preparation of an evaluation study for a complex intervention by substantiating several essential methodological choices. These choices are (A) establishing the most appropriate outcomes and instruments to measure them, including comprehensibility of questionnaires for study participants, (B) exploring the distribution and size of these outcomes in the patient target population and (C) quantifying key assumptions for the sample size calculation. We describe this method through the example of our feasibility study on a person-centered and integrated care (PC-IC) approach for multimorbidity and chronic conditions in general practice.

**Methods:**

In 2021 we conducted a feasibility study in 7 general practices in three regions in the Netherlands. These practices replaced their standard disease management programs for diabetes mellitus type 2, cardiovascular disease, chronic obstructive pulmonary disease, and asthma with the PC-IC approach. Systematically selected questionnaires were administered to eligible patients at baseline and at 6 months, and comprehensibility of the questionnaires was assessed. We defined a composite outcome by comparing different scenarios for combining the questionnaire scores.

**Results:**

The method for thoroughly designing an evaluation study for a complex healthcare intervention consisted of several steps. First, the measurement instruments for the feasibility study were chosen after a structured literature search, consulting experts, checking the questionnaires for comprehensibility by patients, and a consensus meeting with the project team. Next, the questionnaires were applied in the study target population for a period of 6 months. The results were then analysed to explore the distribution and size of these outcomes. Subsequently we assessed the most appropriate outcomes, which led to the creation of a composite outcome in our example. The final step was performing a sample size calculation based on the results of the feasibility study.

**Conclusions:**

Using the described method, we conducted a feasibility study to prepare the evaluation of a complex intervention in Dutch general practice. Our paper is useful for other researchers preparing evaluation studies on complex interventions.

## Introduction

Complex interventions are frequently used at different levels in healthcare. The Medical Research Council (MRC) guidance on developing and evaluating complex interventions states that complex intervention research entails shifting the focus from the ‘binary question of effectiveness’ to whether and how the intervention will be acceptable, implementable, cost-effective, scalable, and transferable across contexts, in order to deliver solutions for real world practice. Part of this process is designing a feasibility study to assess parameters related to the evaluation design and to determine whether and how an intervention can usefully be evaluated [1].

We recently reported on the development of a person-centered and integrated care (PC-IC) approach for multimorbidity and chronic conditions for use at an individual level in general practice [2]. A PC-IC approach in primary care can be considered a complex intervention because of the number of components involved, the range of behaviours targeted, the number of settings involved and the level of flexibility required in applying the intervention [1].

Previously, feasibility studies on complex interventions in general practice have been described. Birke et al [3] and Grant et al [4] both published feasibility studies in general practice based on the MRC guidance, which combined qualitative and quantitative methods. However, they did not elaborate on the process of methodological choices, especially for the quantitative study, and did not mention the effects of their studies on a subsequent evaluation study.

The main aim of this paper is to describe a method for conducting a feasibility study in preparation of an evaluation study by substantiating several essential methodological choices, namely (A) establishing the most appropriate outcomes and instruments to measure them, including comprehensibility of questionnaires for study participants, (B) exploring the distribution and size of these outcomes in the patient target population and (C) quantifying key assumptions for the sample size calculation for the subsequent evaluation study. We will describe this method through the example of our feasibility study on the PC-IC approach for multimorbidity and chronic conditions for use at an individual level in general practice.

## Methods

### Study design

Between March and December 2021, a feasibility study of the recently developed PC-IC approach [2] started in 7 general practices in cooperation with primary care cooperatives in three regions in the Netherlands, i.e. the Nijmegen region (168 general practitioners in total, approximately 290,000 inhabitants, predominantly urban), the Arnhem region (193 general practitioners, ~440,000 inhabitants, predominantly urban) and the Doetinchem region (116 general practitioners, ~150,000 inhabitants, mixed urban/rural). The primary care cooperatives are responsible for organizing a number of single-disease management programs (sDMPs) for chronic conditions in the general practices in their region. They receive funding from health insurers and distribute this among general practices and allied healthcare professionals in their region to provide chronic care. In addition, they organize education and facilitate collaboration with allied healthcare professionals. The primary care cooperatives informed all practices about the study, and the 7 practices volunteered to participate. Practices did not receive financial reimbursement for participation, but were provided with free training in the use of the PC-IC approach. For the feasibility study, these practices replaced their current sDMPs for diabetes mellitus type 2 (DM2), cardiovascular disease (CVD) and chronic obstructive pulmonary disease (COPD) and asthma with the new PC-IC approach for participating patients. The participating practices had not been involved in the previous development of the PC-IC approach. The Research Ethics Committee of the Radboud University Medical Center judged that ethical approval was not required under Dutch National Law (registration number: 2021–8106).

### Intervention

**Figure 1:**
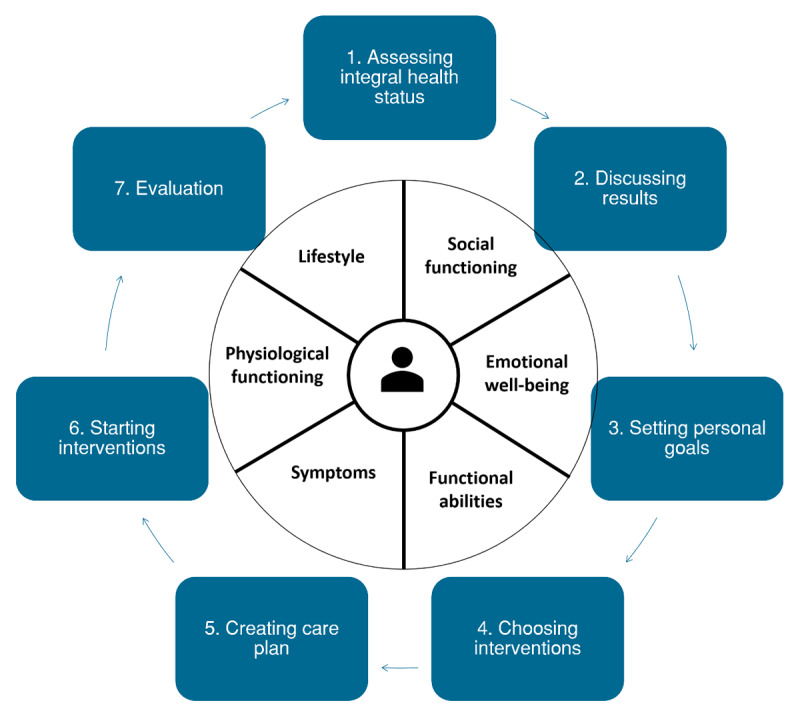
Schematic representation of the Person-Centred and Integrated Care (PC-IC) approach* [2] for the management of patients with chronic diseases and multimorbidity in primary care. * In Step 1 and Step 2 of the PC-IC approach, the Positive Health Tool was implemented. It encompasses six domains: bodily functions, mental and emotional well-being, meaningfulness, quality of life, social well-being/participation, and daily functioning. (4) In Step 1, the patient prepares for the consultation with the healthcare professional by answering questions, preferably digitally. The answers to the questions result in a visual aid (‘Spider Web’), for the discussion between patient and healthcare professional (Step 2). In consultation with the healthcare professional, the patient decides which domain has priority and which personal (health) goals he wants to achieve (Step 3). The healthcare professional discusses with the patient which treatments and support are most suitable for the patient (Step 4) and records the health goals and the chosen treatments (interventions) in a digitalized individual care plan (Step 5) that can be shared with other care providers involved. In Step 6, the selected treatments and other support takes place, after or during which one or more (interim) evaluation visits can be planned, depending on the patient’s needs (Step 7). After a predefined period, for example a year, the patient’s health status is reassessed and the cyclic clinical process repeated.

The intervention was developed in a previous study with patients and healthcare professionals [2]. The core of the PC-IC approach consists of assessing the patient’s health status in a stepwise cyclic process that the healthcare professional completes with the patient (Figure 1). In this process the healthcare professional has the role of case manager who coordinates the care and network around the patient and is the first point of contact for the patient. Given the current organisation of integrated care in the Netherlands, in most general practices practice nurses fulfil this role. The general practitioner remains responsible and can be involved at various points in the cycle, just like other healthcare professionals involved.

Usual care consists of discussing a predefined list of symptoms and laboratory results with the practice nurse. Unlike the PC-IC approach it focusses on the medical domain only, and no personal goals are set. Periodic evaluations are planned according to a predefined schedule that is the same for all patients concerned.

Prior to the feasibility study, general practitioners and practice nurses underwent a certified ‘Positive Health’ training to acquire the necessary knowledge and skills to integrate the new PC-IC approach into their consultations, i.e. interpreting and discussing the Positive Health Tool.

### Participant selection

Each participating primary care cooperative recruited two to three practices to implement the new PC-IC approach and to enrol eligible patients. Patients were eligible for inclusion in the new PC-IC approach if: diagnosed with CVD and/or being managed for its risk factors through cardiovascular risk management (CVRM), DM2, COPD and/or asthma; received disease management in a sDMP; and was willing to provide written informed consent. Exclusion criteria were complete lack of command of the Dutch language, even with the help of a relative; and terminal illness with a life expectancy <3 months.

Depending on a practice’s usual workflow, patients were contacted by practice support staff through telephone or e-mail before a scheduled consultation to provide information about the new PC-IC approach and the feasibility study. We instructed practices to ask all eligible patients that were already scheduled for an appointment in the upcoming months to participate in the study chronologically and aimed to include up to 25 patients per practice. Practices were asked to include at least two patients with a lower educational level (having completed no more than post-secondary vocational education level 1 (or equivalent) education) in the study to ensure a representative and inclusive sample, as this group is often underrepresented in research and more at risk for (multiple) chronic diseases [5,6]. Patients did not receive financial compensation for their study participation. We recorded data on inclusion and retention rate of participants.

### Selection of outcomes and measurement instruments

When preparing the feasibility study we first defined the desired outcome domains to be measured at patient level, in accordance with the relevant quadruple aims (i.e. better patient experience, and better population health) [7]. For the purpose this paper, namely presenting an example of a thorough preparation of an evaluation study, we have chosen to omit the preparation of the other two quadruple aims (i.e. better healthcare provider experience and better cost-effectiveness). Next, we traced potential measurement instruments (i.e. questionnaires) for the selected patient-related outcomes by searching literature databases for previously used questionnaires and by consulting experts. We searched PubMed and Google Scholar with MeSH and tiab terms: ‘surveys and questionnaires’, ‘Population Health’, ‘Health-related quality of life’, ‘Patient satisfaction’, ‘Patient experience’, ‘General Practice’ and ‘Chronic Diseases’, in multiple combinations. We generated a longlist of suitable questionnaires, their psychometric characteristics, and their (dis)advantages. In a consensus meeting the project team selected the final questionnaires to be tested in the feasibility study from the longlist. Selection criteria were: questionnaire is widely used, validated, available in Dutch and consists of less than 20 questions. Table 1 provides an overview of the candidate questionnaires for the study per outcome for the population health and patient experience domains.

**Table 1:** Longlist of suitable questionnaires; questionnaires written in *italic* typeface were chosen in a consensus meeting of the project team to be used in the feasibility study.


POPULATION HEALTH					

OUTCOME	QUESTIONNAIRE	AUTHORS/SOURCE	DUTCH VALIDATED VERSION	NUMBER OF QUESTIONS	SCORING RANGE*

**Quality of Life**	*Satisfaction with Life Scale (SWLS)*	Diener, 1985 [8].	*Yes*	*5*	*5–35*

	*Life Satisfaction Questionnaire (LSQ)*	Fugl-Meyer, 1991 [9].	*Yes*	*9*	*1–6*

**Health state**	*EQ-5D-5L*	Herdman, 2011 [10].	*Yes*	*6*	*–0.446–1*

	SF12	Ware, 1996 [11].	Yes	12	0–100

**Functioning and participation**	ICF Primary Care instrument	Postma, 2018 [12].	Yes	73	1–5^#^

**Self-management**	*Patient Activation Measure (PAM)*	Hibbard, 2004 [13].	*Yes*	*13*	*0–100*

**Health related quality of life**	*PROMIS global 10*	Hays, 2009 [14].	*Yes*	*10*	*T-score*

**Well-being**	*Well-being of Older People (WOOP)*	Hackert, 2021 [15].	*Yes*	*9*	*9–45*

**Well-being**	ICE-CAP-O	Makai, 2013 [16].	Yes	5	0–1.00

**PATIENT EXPERIENCE**					

**OUTCOME**	**QUESTIONNAIRE**	**AUTHORS/SOURCE**	**DUTCH VERSION**	**NUMBER OF QUESTIONS**	SCORING RANGE*

**Chronic care**	Patient Assessment of Care for Chronic Conditions (PACIC)	Glasgow, 2005 [17].	Yes	20	1–5

**Quality of primary care**	Consumer Quality Index (CQ-index) Daytime General Practice Care	Meuwissen, 2009 [18].	Yes	53	0–10

**Patient experience of chronic care in primary care**	PREM Chronic care	Hendriks, 2016 [19].	Yes	25	Net promotor score

**Person centred coordinated care experiences**	*Person Centred Coordinated Care Experiences Questionnaire (P3CEQ)*	Lloyd, 2019 [20].	*Yes*	*11*	*0–30*

**Person centred care experience**	Person Centred Primary Care Measure (PCPCM)	Etz, 2019 [21].	No	11	8–44

**Continuity of care**	Nijmegen Continuity Questionnaire (NCQ)	Uijen, 2011 [22].	Yes	30	1–5


* Higher score is better. # Higher score is worse.

The questionnaires selected from the longlist by the project team (*italic* typeface in Table 1) were applied to the included patients from the participating practices at baseline and at 6 months follow-up, either through Castor Electronic Data Capture (a digital secured clinical data management platform) or on paper, depending on a patient’s preference. In the 6-month questionnaire we asked patients about their experience with completing the respective questionnaires using closed and open-ended questions.

We asked healthcare professionals to record the number and duration of consultations with their included patients, and the general goals and interventions as recorded in included patients’ care plans. Data on patient characteristics were extracted from the patients’ electronic medical files at 6 months follow up.

#### Comprehensibility of questionnaires

To ensure that every patient, regardless of health literacy, could participate and to obtain data that represented the entire population, we considered it crucial that all patients could understand the questionnaires. Therefore, the questionnaires selected for the feasibility study were assessed on comprehensibility by Pharos, the Dutch expertise centre for Health Disparities, using their checklist to access and enhance the comprehensibility and accessibility of questionnaires for adults with low health literacy (‘Pharos Checklist for Questionnaires in Healthcare’) [23]. The checklist consists of four components: 1) comprehensibility, 2) accessibility, 3) layout, and 4) validation. In our study we focused on comprehensibility, accessibility, and language level. We studied this with the checklist, with the computer tool ‘Resounding Language’ [24] – which indicates the language level according to the Common European Framework of Reference for Languages (CEFR A1-C2) [25] – and also by think aloud sessions with patients with limited literacy skills [26].

### Data analysis

Patients’ responses to the questionnaire items were analysed by comparing the baseline scores with those measured at 6 months. Patient characteristics and outcomes at baseline and at 6 months follow-up were described as medians with interquartile range (IQR) or as means with standard deviation (SD) in case of continuous variables, and as frequency with percentage in case of categorical variables. Missing values in outcome measurements were imputed by entering the average value of the scores on the other questions in the questionnaire. To test for statistically significant differences between the outcomes at baseline and at 6 months we used paired t-tests and Wilcoxon signed rank tests, depending on the distribution of the variable. A two-sided p-value < 0.05 was considered to be statistically significant. Because anchor-based calculations to define minimal clinically important difference (MCID) were unavailable for most questionnaires in the context of our study population and using different methods for different questionnaires could create bias in the analysis, we chose to use a distribution-based method to define MCIDs for all selected questionnaires, by using a definition of 0.5 times the SD of the baseline mean score [27]. We analysed the data on duration of consultations and goals and interventions as recorded in the care plans using descriptive statistics. All statistical analyses were carried out using SPSS (IBM Corp. Released 2023. IBM SPSS Statistics for Windows, Version 29.0.2.0 Armonk, NY: IBM Corp).

Necessary information for the sample size calculation of the forthcoming evaluation study to be derived from the analysis included retention rate of patients and estimates of effect sizes of the selected questionnaires.

### Composite outcome

After reviewing the questionnaire results, the project team deliberated on the primary outcome to use for the forthcoming evaluation study. Due to the complexity of our intervention and to ensure statistical efficiency [28] we wanted to combine the two patient-related aims of the quadruple aims concept [7] which we consider to be of equal clinical importance. These outcomes are health-related quality of life, also known as a Patient Reported Outcome Measure (PROM), and patient experience of care, also referred to as a Patient Reported Experience Measure (PREM). We use a resulting composite outcome as the primary outcome in the evaluation study. We defined the composite outcome by assessing and comparing different scenarios of combinations of improvement on different questionnaires scores.

## Results

### Study participants

**Figure 2:**
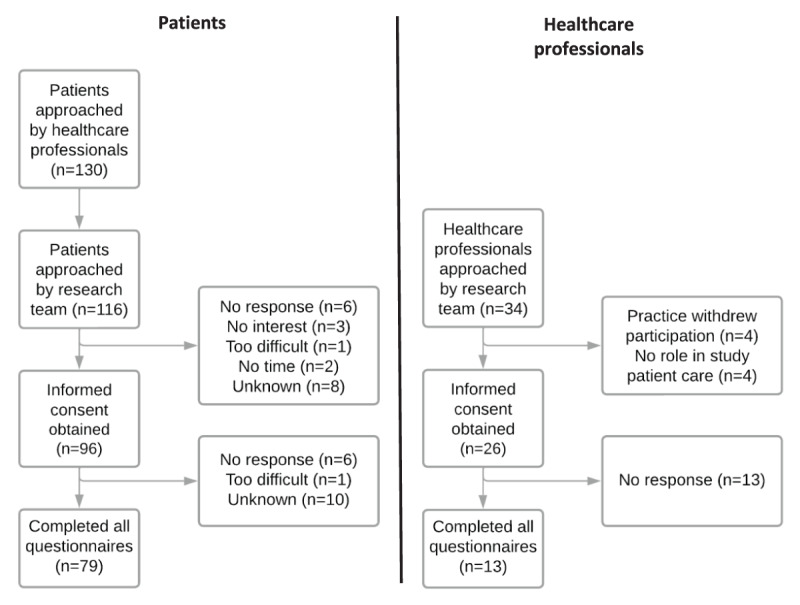
Flowchart of inclusion of patients and healthcare professionals.

In total, 26 healthcare professionals and 96 patients from the 7 general practices participated in the feasibility study (Figure 2). Practices invited a median of 14 patients (range 10–31) and median inclusion was 11 patients (range 5–24) per practice. At 6 months follow-up, 79 (82%) patients had completed the questionnaires. Table 2 shows the baseline characteristics of the participating patients and healthcare professionals. The mean (SD) age of the patients was 61.6 (12.6) years (range 21–83). Sixty-two percent of the patients was female, and the educational level was classified as 38% “low”, 30% “middle” and 31% “high”. One patient was lost during follow-up before we were able to obtain data on medication use and chronic diseases. Most patients (n = 86, 91%) were included in the CVD/CVRM sDMP, 34% in the COPD/asthma sDMP, and 54% in the DM2 sDMP. Twenty-four patients (25%) were included in two sDMPs. Mean (SD) follow-up time was 166 (39) days. Of the 96 patients who completed the baseline questionnaires, 94 (98%) received at least 1 PC-IC consultation during the 6-month follow-up, of which the majority (73%) also received a second consultation (appendix B). In 77 patients an individual care plan was created and added to the patient’s medical record.

**Table 2:** Characteristics of the patients and healthcare professionals participating in the feasibility study.


PATIENTS	NUMBER (%) (n = 96)

Sex, male	36 (38%)

Age, ≥65 years	48 (50%)

Paid work, yes	37 (39%)

**Educational level** ^1^	

Low	36 (38%)

Middle	28 (30%)

High	30 (31%)

Limited literacy^2^	21 (22%)

**Lifestyle**	

Alcohol consumption, average of ≥1 glass/day	28 (29%)

Active smoking, yes	21 (22%)

Exercise, ≥5 days per week^3^	47 (49%)

Polypharmacy, chronic use of ≥5 different medications^4^	47 (49%)

≥3 chronic diseases, yes	50 (52%)

**sDMP inclusion**	

COPD/asthma, yes	32 (33%)

CVRM, yes	87 (91%)

Diabetes, yes	43 (45%)

Included in 2 sDMPs^5^	24 (25%)

**HEALTHCARE PROFESSIONALS**	**NUMBER (%) (n = 26)**

Sex, male	5 (19%)

Practice nurses	13 (50%)

General practitioners	13 (50%)

5 or more years relevant working experience	18 (69%)

Healthcare professionals	Number (%)(n = 26)

**Type of practice**	

Solo practice	8 (31%)

Group practice	12 (46%)

Practice integrated in multidisciplinary healthcare center	6 (23%)


^1^ Educational level was determined based on the highest completed education, creating 3 groups: High: higher vocational or university education, Middle: higher secondary general education or post-secondary vocational education, Low: all other types of education. ^2^ Patients who reported to ever have experienced problems with reading medical flyers or information. ^3^ Based on the Dutch exercise guideline [29] that states to exercise at least 5 days a week for at least 30 minutes per session. ^4^ Polypharmacy is defined as the chronic use of 5 or more different medications [30,31]. We excluded dermatological medications, ferrous fumarate, artificial tears, vitamins, and fibres. Other eye drops were included if they were chronically used. Medications were included in chronic use if they were prescribed for ≥90 days. Combination preparations were counted as 2 separate medications. ^5^ The combination Diabetes sDMP and CVD/CVRM sDMP was not included as patients with both DM2 and CVD or elevated cardiovascular risk are only treated in the sDMP for DM2.

Of the participating healthcare professionals, 81% were female. Fifty percent were practice nurses and 50% were general practitioners. Approximately two-thirds (69%) had 5 or more years of experience in their current profession. Most healthcare professionals (46%) worked in a group practice (i.e. a practice run by two or more general practitioners).

### Analysis of patient-level questionnaires

Table 3 shows the median (IQR) and mean (SD) scores on the questionnaires at baseline and the difference between the baseline and 6-month follow-up measurements. It also shows the percentage of people who showed an MCID at 6 months. None of the questionnaires showed a statistically significant change at 6 months follow up. The number of patients who showed a positive MCID was most apparent for the EQ-5D-5 L VAS (n = 19 patients), PAM (n = 16) and PROMIS-GPH (n = 18). A negative MCID was most prevalent for the LSQ (n = 17), P3CEQ (n = 20), PROMIS-GMH (n = 17) and the WOOP (n = 18). The rightmost column shows how many patients had difficulty with answering the questions per questionnaire. Patients found the SWLS and the P3CEQ questionnaires the most difficult to complete, although for different reasons: according to the feedback obtained the SWLS was hard to understand and confronting, while not all questions of the P3CEQ were applicable to every participant. A sub analysis for the patients with a lower educational level can be found in Appendix C. Results from this sub analysis were similar to the total study population, with the same questionnaires which showed the highest number of participants with positive and negative MCIDs and the most difficulty with understanding the SWLS and P3CEQ. Difficulty with the questionnaires was not more prevalent in patients with a lower educational level.

**Table 3:** Scores on the selected questionnaires for the outcomes ‘patient experience’ and ‘population health’ in 96 patients with one or more chronic conditions. Questionnaires marked *italic* were selected for the forthcoming evaluation study.


QUESTIONNAIRE	COMPLETED QUESTIONNAIRE AT BASELINE (n)	COMPLETED QUESTIONNAIRE AT 6 MONTHS FOLLOW UP ^+^ (n)	MEDIAN (IQR) SCORE AT BASELINE	MEAN (SD) AT BASELINE	MEAN CHANGE	p-VALUE	MCID†	n (%) NEGATIVE MCID	n (%) NO CHANGE	n (%) POSITIVE MCID	n (%) DIFFICULTY WITH QUESTIONS (n = 86)

EQ-5D-5L index	96	79	0.85 (0.1)	0.83 (0.19)	–0.01	0.470*	0.10	13 (16.5)	58 (73.4)	8 (10.1)	8 (9.3)

EQ-5D-5L visual analogue scale (VAS)	96	79	80 (20)	77.49 (16.02)	1.87	0.09*	8.01	11 (13.9)	49 (62.0)	19 (24.1)	8 (9.3)

Life Satisfaction Questionnaire (LSQ)	91	79	5 (0.7)	4.97 (0.59)	–0.02	0.567*	0.30	17 (22.7)	43 (57.3)	15 (20.0)	10 (11.6)

*Person-Centred Coordinated Care Experience Questionnaire (P3CEQ)*	*96*	*79*	*14.0 (9.8)*	*12.65 (6.19)*	*–0.23*	0.924*	*3.10*	*20 (25.3)*	*40 (50.6)*	*19 (24.1)*	*18 (20.9)*

*Patient Activation Measure (PAM)*	*88*	*72*	*62.5 (19.8)*	*64.63 (14.62)*	*0.62*	0.394*	*7.31*	*11 (16.7)*	*39 (59.1)*	*16 (24.2)*	*15 (17.4)*

*PROMIS–general physical health (GPH)*	*96*	*79*	*45.7 (11.3)*	*45.82 (7.90)*	*0.96*	*0.071^#^*	*3.95*	*10 (12.7)*	*51 (64.6)*	*18 (22.8)*	*6 (7.0)*

*PROMIS–general mental health (GMH)*	*96*	*79*	*45.3 (8.3)*	*46.74 (6.64)*	*–0.50*	0.843*	*3.32*	*17 (21.5)*	*49 (62.0)*	*13 (16.5)*	*6 (7.0)*

Satisfaction with Life Scale (SWLS)	96	79	29 (9.8)	26.80 (6.82)	–0.09	0.997*	3.41	14 (17.7)	51 (64.6)	14 (17.7)	17 (19.8)

Well-being of Older People (WOOP)	96	79	37.1 (5.1)	37.07 (4.49)	–0.27	0.290*	2.25	18 (22.8)	50 (63.3)	11 (13.9)	5 (5.8)


^+^Statistical tests were only performed on paired values of 79 participants who completed all questionnaires. Baseline characteristics were similar to the group of 96 patients. *Wilcoxon test; † MCID defined as 0.5 × SD of the baseline questionnaire score; # Paired t-test. MCID = minimal clinically important difference; PROMIS = patient reported outcomes measurement information system; SD = standard deviation.

#### Comprehensibility

Based on the Pharos Checklist for Questionnaires in Healthcare assessment, the Resounding Language software application, and the think aloud sessions, most of the questionnaires were written in language level B1 according to the Common European Framework of Reference and comprehensible. According to the Pharos Checklist for Questionnaires in Healthcare, the most challenging aspects of the questionnaires were: the number of response options for each question (often more than 4 options), the questionnaire layout which was considered to be too complicated, and the use of abbreviations. In addition, the questionnaires by themselves were not considered too long, mostly less than 20 questions, but all the questionnaires together were considered too lengthy, especially for subjects with limited reading and writing skills. The EQ-5D-5 L, PAM and PROMIS Global 10 tested best on comprehensibility, see appendix A for more details.

### Selection of questionnaires

As we aimed to limit the burden of completing questionnaires for participants in the subsequent evaluation study, the project team decided to select for the composite primary outcome one patient experience questionnaire (the P3CEQ) and one health-related quality of life questionnaire (the PROMIS global 10), which has 2 domain scores, namely the General Mental Health and General Physical Health. This was based on three criteria: the fewest patients reporting difficulty with these questionnaires; positive testing of the comprehensibility; and the largest number of participants scoring either a positive or negative MCID compared to the other questionnaires, thus demonstrating sensitivity of the questionnaire. In addition, we added the PAM, which is an intermediate outcome with predictive value for improved health outcomes, experience of care and decreased costs of healthcare as a secondary outcome [32].

### Definition of composite primary outcome for the evaluation study

Due to the complexity of our PC-IC intervention, the fact that we value each of the patient-level quadruple aims (i.e., population health and patient experience) to be equally important, and to ensure statistical efficiency we chose a combination of three outcomes to serve as the composite primary outcome for the forthcoming evaluation study. In this step we therefore combined the scores of the P3CEQ and the PROMIS global 10 General Physical Health and General Mental Health into one composite primary outcome for the evaluation study. Table 4 shows different scenarios for defining the composite outcome. Only 4 patients showed a clinically important improvement on all three questionnaire scores and 8 patients did so on at least two scores without a decrease in the other score. Twenty-four patients improved on at least one of the scores, without a decrease in the other scores. Because of the observed discrimination between the different scenarios and the fact that we did not want to allow a decrease in one score to contribute to a positive overall composite outcome, the final definition of the composite outcome for the evaluation study is: the MCID improves on at least one of the questionnaire scores, without a decrease in the other scores. Thus, the final definition of the composite primary outcome consists of the percentage of patients who have a clinically relevant improvement of at least the MCID in either the PROMIS GMH score *OR* the PROMIS GPH score *OR* the P3CEQ-score *AND* no decrease of at least the MCID in either the PROMIS GMH score *AND* the PROMIS GPH score *AND* the P3CEQ-score.

**Table 4:** Different scenarios for the composite primary outcome for the evaluation study.


QUESTIONNAIRE:	n (%)

PROMIS GPH; MCID improved	18 (22.8)

PROMIS GMH; MCID improved	13 (16.5)

P3CEQ; MCID improved	19 (24.1)

Scenarios for combining the three questionnaires* to define the composite primary outcome:	

MCID improved of at least one (decrease allowed)	37 (46.8)

MCID improved of at least one (no decrease allowed)	24 (30.4)

MCID improved of at least two (decrease allowed)	9 (11.4)

MCID improved of at least two (no decrease allowed)	8 (10.1)

MCID improved of all three questionnaires	4 (5.1)


* PROMIS GPH & GMH & P3CEQ; GPH = general physical health; GMH = mental health; MCID = minimal clinically important difference; P3CEQ = Person Centred Coordinated Care Experiences Questionnaire; PROMIS = patient reported outcomes measurement information system.

#### Sample size calculation for the evaluation study

Finally, we performed a sample size calculation for the evaluation study (a cluster randomized controlled trial) based on the results of the feasibility study. Of the 79 patients who completed all questionnaires we found that 30.4% met the requirements for a positive composite primary outcome. Loss to follow-up during the 6-month observation period was 18%. Based on these results and the longer duration of the intervention during the follow up in the evaluation study (12 months versus 6 months in the feasibility study) we anticipated that in the evaluation study 35% of the intervention patients compared to an estimated 20% of the usual care patients will improve on the composite outcome. We estimated that for the evaluation study we need a total sample size of at least 680 patients (i.e., a total of 17 general practices with an average of 40 included patients per practice) to detect a difference of 15% in the composite primary outcome between the PC-IC and control groups, taking into account an alpha of 5%, a power of 80%, an intra-class correlation coefficient of 0.03 [33], and a loss to follow-up of 20% (clinicaltrial.gov ID NCT05495230).

## Discussion

### Summary of results

With the feasibility study reported in this paper we aimed to describe a method for preparing an evaluation study for management of patients with chronic conditions by substantiating several essential methodological choices, which included selecting the most appropriate and most comprehensible outcome measurements and creating a composite outcome, as is recommended when evaluating a complex intervention such as our previously developed PC-IC approach [1].

The method consists of several steps. First, choosing suitable measurement instruments for the feasibility study through structured literature search, consulting experts, and checking the questionnaires for comprehensibility by patients. Next, applying the selected questionnaires in the study target population for a period of 6 months, analysing the distribution, size and appropriateness of the measured outcomes and creation of a composite outcome. The final step was performing a sample size calculation for the subsequent evaluation study based on the results of the feasibility study.

### Comparison to previous research

The design of the feasibility study is in line with the MRC guideline on designing and evaluating complex interventions [1]. Other mixed methods feasibility studies preparing the evaluation of a complex intervention have been reported [3,4], but none described the process in detail. These authors have mostly combined the reporting of both qualitative and quantitative results from their feasibility study in a single paper. We chose to report our feasibility study in two papers, because of two separate aims of the study. The first of the two papers describes the experience of patients with the PC-IC intervention [34], while the current paper focusses on preparing the evaluation study.

We chose to define a composite outcome based on the data from the feasibility study. The main reasons for using a composite outcome are to avoid an arbitrary choice between several important outcomes and statistical efficiency [28]. In addition, from our data is it apparent that although the group mean scores did not significantly change, individual participants did improve on important outcome measures, which makes the composite outcome as defined more suitable for the evaluation study of a person-centered approach. Arguments against a composite outcome might be that it can be misleading, that (some of its) components can be irrelevant to (some) patients or be clinically irrelevant, and that it might be less transparent due to possible post-hoc changes in the definition of the outcome [35]. However, because the composite outcome we compiled is based on empirical data from the feasibility study and in line with previously defined aims for health care [7], this composite outcome is relevant for patients and healthcare providers. In addition, we adhered to the standards for using a composite outcome, by only combining components of similar clinical importance and taking care to define them consistently throughout the paper. In the evaluation study we will analyse the prespecified composite outcome, but will also list the results for its separate components [35].

### Strengths and limitations

The main strength of the method described in our paper is the rigorous preparation of evaluation study for three reasons. The first is reduction of the study burden on the larger trial population by testing the questionnaires in a smaller patient sample and selecting the most appropriate questionnaires. The second strength is the careful selection of the questionnaires to be used based on thorough literature review, testing for comprehensibility, and expert consensus. The third and final strength is the creation of the composite outcome that is based on empirical data and well-defined before the evaluation study, including person-centred outcomes by using MCIDs instead of population-based means. Having multiple questionnaires, we were faced with a choice on how to best define minimal clinically important difference for all questionnaires. Because anchor-based calculations were unavailable for most questionnaires in the context of our study population and using different methods for different questionnaires could create bias in the analysis, we chose to use a distribution-based method for all selected questionnaires. Distribution-based methods do not fully cover the issue of clinical importance, but as Sloan stated “there are many methods available to ascertaining an MCID, none are perfect, but all are useful” [36].

There are several important limitations that also deserve consideration. One is the limited duration of follow up (6 months), in which we did not expect to see substantial improvement in patient-related outcomes, because usually a longer follow up period is necessary to find the effect of behavioural change and complex interventions. Another clear limitation is that, at the time of the feasibility study, there were no instruments available to measure patient experience other than the P3CEQ, which fulfilled our selection criteria, i.e. instrument is widely used, validated, available in Dutch and consists of less than 20 questions, and therefore we could not test other questionnaires for this aim.

### Implications for our evaluation study

A cluster randomized controlled trial (clinicaltrial.gov ID NCT05495230) is planned to evaluate the PC-IC approach, which is based on the elaborate preparation in the feasibility study as reported in this paper. Our paper can be useful for other researchers preparing (cluster) randomized controlled trials on complex interventions by serving as an example of the different preparatory steps and considerations.

Given the newly defined quintuple aim [37], which includes advancing health equity, and the differences between the overall population and patients with lower educational levels in our qualitative study [34], we applied for and received additional funding. This funding allows us to further investigate and refine the PC-IC approach for this subgroup, including additional testing of questionnaires, adapting recruitment and dissemination strategies, and evaluating effects in a separate evaluation study (clinicaltrial.gov ID NCT05972031).

## Conclusion

This paper aimed to describe a method to thoroughly prepare the evaluation of a complex intervention by substantiating several essential methodological choices, using the feasibility study on a new PC-IC approach in Dutch general practice as an example. Our paper can be useful for other researchers preparing (cluster) randomized controlled trials on complex interventions.

## Additional File

The additional file for this article can be found as follows:

10.5334/ijic.8998.s1 Appendix. Appendix A–Appendix C.
