# Supplementary material for: Optimizing Study Design for Evaluating Complex Interventions: An Example of a Feasibility Study in Person-Centered and Integrated Chronic Disease Care in Dutch General Practice
Source: Int J Integr Care. 2025 Nov 6;25(4):6. doi: 10.5334/ijic.8998 (PMC12594077; doi:10.5334/ijic.8998)
Supplement: Appendix. — Appendix A–Appendix C. [file ijic-25-4-8998-s1.pdf]

Appendix

Appendix A. Details on PCQH and comprehensibility of questionnaires.

In the PCQH the eight domains that make up comprehensibility of a questionnaire are: 1) language level; 2) existence of a brief and understandable instruction; 3) number of questions; 4) number of response options; 5) medical terminology and acronyms; 6) specific queries; 7) statements; and 8) questions in the active voice. A color-coded scale is used to score each domain; green indicates "optimal" adherence to the domain standards, orange indicates "acceptable" adherence, and red indicates "significant inadequacies" that call for considerable improvements for improved accessibility and clarity.

Questionnaire	
Satisfaction with Life Scale (SWLS)	The many (7) answer options make this a difficult questionnaire. Also, the wording (e.g., "rather") makes it difficult to answer correctly, in addition to the many answer options. The questions are fairly easy to understand. Language level B1 (clear language, short questionnaire of 5 questions).
Life Satisfaction Questionnaire (LSQ)	This questionnaire is not understandable due to the difficult answer categories. It also does not seem feasible to administer orally. During testing, it was not possible to explain it.
EQ-5D-5L	Not tested, but has an NRS scale, so no difficulties are expected.
Patient Activation Measure (PAM)	This questionnaire is easy to complete, especially orally. Not many difficult words are used. Language level B1. Layout: For written completion, please place the answer options under the question.

	Write out abbreviations.
PROMIS global 10	The PROMIS 10 is easy to understand orally, except for questions 4, 5, and 6, which require more explanation. The answer scales keep changing, which makes it more difficult. Therefore, placing the answer options under the question prevents confusion. Written completion would be easier if the questions were not in a table, but instead written out with the answer options listed below. Language level: B1.
Well-being of Older People (WOOP)	Despite language level B1, the language ambassador finds this a difficult questionnaire; the answer options are difficult due to language usage, and the way questions are posed is challenging.
ICE-CAP-O	Not tested.
Patient Assessment of Care for Chronic Conditions (PACIC)	Not tested.
Consumer Quality Index (CQ-index) Huisartsenzorg Overdag	Not tested.
PREM Chronische zorg	Available in simple language, not validated.
Person Centred Coordinated Care Experiences Questionnaire (P3CEQ)	Language level B1, not tested with language ambassador.
Person Centred Primary Care Measure (PCPCM)	Adapted in another study at Radboudumc to a more understandable version, but not yet validated.
Nijmegen Continuity Questionnaire (NCQ)	Not tested.

Appendix B. Details on process outcomes.

**Patients**	Number (%) N=96
At least 1 PC-IC consultation	94 (97.9)
At least 2 PC-IC consultations	68 (70.8)
At least 3 PC-IC consultations	32 (33.3)
At least 4 PC-IC consultations	2 (2.1)
Individual care plan present	77 (81.1)
At least 1 referral	17 (17.9)
Referral to specialized care in hospital	3 (3.2)
Referral to mental health care	4 (4.2)
Referral to dietician or lifestyle coach	4 (4.2)
Referral to physiotherapy	4 (4.2)
Referral to social care	3 (3.1)

Appendix C. Sub analysis in patients with low educational level

Scores on the selected questionnaires for the outcomes ‘patient experience’ and ‘population health’ in 36 patients with one or more chronic conditions and low educational level. Questionnaires marked *italic* were selected for the forthcoming evaluation study.

**Questionnaire**	**Completed questionnaire at baseline (n)**	**Completed questionnaire at 6 months follow up[+] (n)**	**Median (IQR) score at baseline**	**Mean (SD) at baseline**	**Mean change**	p-value	**MCID†**	**n (%) negative MCID**	**n (%) no change**	**n (%) positive MCID**	**n (%) difficulty with questions (n=31)**
EQ-5D-5L index	*36*	*26*	*0.85 (0.2)*	*0.81 (0.20)*	*-0.01*	*0.520**	*0.10*	*4 (15.4)*	*18 (69.2)*	*4 (15.4)*	*2 (6.5)*
EQ-5D-5L VAS	*36*	*26*	*80 (29)*	*75.47 (18.10)*	*4.04*	*0.138**	*8.01*	*3 (11.5)*	*14 (53.8)*	*9 (34.6)*	*2 (6.5)*
LSQ	35	26	5 (0.7)	4.96 (0.58)	-0.01	0.731*	0.30	7 (26.9)	14 (53.8)	5 (19.2)	4 (12.9)
P3CEQ	*36*	*26*	*13.7 (8.9)*	*11.92 (7.01)*	*-0.53*	*0.819**	*3.10*	*7 (26.9)*	*14 (53.8)*	*5 (19.2)*	*6 (19.4)*
PAM	*33*	*26*	*59.1 (17.0)*	*62.84 (14.68)*	*1.94*	*0.619**	*7.31*	*2 (10.5)*	*12 (63.2)*	*5 (26.3)*	*4 (12.9)*

PROMIS – GPH	*36*	*26*	*45.4 (10.8)*	*44.72 (7.90)*	*1.72*	*0.061*[#]	*3.95*	*2 (7.7)*	*16 (61.5)*	*8 (30.8)*	*2 (6.5)*
PROMIS - GMH	*36*	*26*	*44.8 (4.6)*	*45.09 (4.62)*	*-0.58*	*0.428*[#]	*3.32*	*4 (15.4)*	*20 (76.9)*	*2 (7.7)*	*2 (6.5)*
SWLS	*36*	*26*	29.5 (10.8)	26.97 (7.03)	-1.04	0.958*	3.41	*6 (23.1)*	*16 (61.5)*	*4 (15.4)*	*6 (19.4)*
WOOP	*36*	*26*	36.6 (6.0)	36.09 (4.44)	0.56	0.237*	2.25	2 (7.7)	19 (73.1)	5 (19.2)	2 (6.5)

[+]statistical tests were only performed on paired values of 79 participants who completed all questionnaires. Baseline characteristics were similar to the group of 96 patients. *Wilcoxon test; † MCID defined as 0.5 x SD of the baseline questionnaire score of total population; # Paired t-test. GPH = general physical health; GMH = general mental health; LSQ = life satisfaction questionnaire; MCID = minimal clinically important difference; P3CEQ = Person Centred Coordinated Care Experiences Questionnaire; PAM = patient activation measure; PROMIS = patient reported outcomes measurement information system; SD = standard deviation; SWLS = satisfaction with life scale; VAS = visual analogue scale; WOOP = well-being of older people.
